# An Investigation on Workplace Violence in an Infectious Disease Hospital: A Mixed-Methods Study from the Perspectives of Healthcare Workers and Patients

**DOI:** 10.3390/ejihpe15080155

**Published:** 2025-08-11

**Authors:** Yuting Tang, Min Zhang, Chuning He, Yiming Huang, Xinxin Fang, Xuechun Wang, Fuyuan Wang, Yiran Zhang

**Affiliations:** School of Population Medicine and Public Health, Chinese Academy of Medical Sciences & Peking Union Medical College, Beijing 100730, China; tyting0109@student.pumc.edu.cn (Y.T.); hechuning@student.pumc.edu.cn (C.H.); huangyiming@sph.pumc.edu.cn (Y.H.); s2023027004@pumc.edu.cn (X.F.); wangxuechun@student.pumc.edu.cn (X.W.); wangfuyuan@student.pumc.edu.cn (F.W.); s2024027025@student.pumc.edu.cn (Y.Z.)

**Keywords:** workplace violence, healthcare workers, mixed-methods research, infectious disease hospital, patient perspective, CARE framework

## Abstract

Workplace violence (WPV) against healthcare workers (HCWs) in infectious disease hospitals, particularly in high-risk settings, remains a critical yet understudied occupational hazard. A mixed-methods study was conducted at a Chinese infectious disease hospital, combining quantitative surveys (N = 675) and semi-structured interviews (28 HCWs, 17 patients/families). Logistic regression was used to analyze WPV incidence and predictors, while a thematic analysis of interview data identified contextual drivers. Psychological violence (34.1%) was significantly more prevalent than physical violence (2.2%), with outpatient departments (44.1%) and temporary staff (OR = 1.72) at the highest risk. Qualitative themes revealed systemic triggers, including communication breakdowns, environmental stressors, and organizational gaps in safety protocols and reporting. This study addressed a critical gap by incorporating the perspectives of HCWs and patients, revealing that WPV perceptions vary due to differing expectations and experiences. From these results, the CARE (Communication, Advocacy, Respect, Education) framework proposes actionable strategies: standardized SBAR communication protocols, enhanced security protocols in high-risk units, and mandatory anti-violence training. These findings underscore the need to strengthen the occupational health system to mitigate WPV and to improve healthcare quality.

## 1. Introduction

Workplace violence (WPV) is an increasingly prominent issue in the professional environment of healthcare workers (HCWs), becoming a global problem that needs to be solved urgently and one of the main occupational hazards they face (Spelten et al., 2020b). According to the World Health Organization (WHO), up to 38% of health workers experience physical violence at some point in their careers, leading to psychological distress and burnout, which compromises healthcare quality (World Health Organization, 2022). The International Labour Organization (ILO) Convention No. 190 on Violence and Harassment (Violence and harassment convention, No. 190) integrates “violence and harassment” into a single comprehensive concept, recognizing it as an independent and complete category. “Violence and harassment” refers to a series of unacceptable acts, practices, or threats that may intentionally cause physical, psychological, sexual, or economic harm, whether they occur once or repeatedly, including sex-based violence and harassment (International Labour Office, 2019a, 2019b). WPV is a serious threat to the safety and health of workers in all sectors. It includes physical and psychological violence, which may take the form of murder, physical assault, verbal abuse, threats, sexual harassment, bullying, and cyber violence (Chappell & Di Martino, 2006).

In a systematic review, WPV was shown to affect 38% of HCWs globally (World Health Organization, 2022), especially in emergency/psychiatry departments (Hills & Joyce, 2013). Previous studies (2008~2019) (Estryn-Béhar et al., 2008; Farrell et al., 2014; Gillespie et al., 2017; Li et al., 2020) showed that department, gender, occupation, operating environment, and work–rest schedule systems were associated with WPV, but these factors might vary by country/region. However, WPV in healthcare institutions was severely under-reported (Shafran-Tikva et al., 2017b), particularly psychological violence (Acququadro Maran et al., 2018). Some studies explained the reasons for the under-reporting of violence (Ferri et al., 2016): guilt or a sense of shame, fear of being blamed or reprisals by the accused (Copeland & Henry, 2017), lack of time and unwillingness to fill out forms (Arnetz, 1998), and concerns about the consequences (Lafta & Falah, 2019). Other reasons might be that some HCWs identify violence as a part of their job (Baig et al., 2018) and that reporting violence carried out by patients to the organization was perceived as a conflict of duty (Williamson et al., 2014). It was also assumed that management will not take any protective or corrective action (Copeland & Henry, 2017; Lafta & Falah, 2019).

Based on the international consensus that WPV impacts individuals, organizations, and societies, Zhang introduced this framework into China and promoted its implementation through policy advocacy, institutional training, and systematic integration into occupational health frameworks (Zhang, 2019). A review of 68 studies found that physical, psychological, and emotional symptoms and work impacts were the most common and significant consequences of WPV (Lanctôt & Guay, 2014), which could profoundly disrupt the lives of workers and had serious financial consequences, such as lost income and increased medical costs (Papa & Venella, 2013; Hassard et al., 2019; Maguire et al., 2018). For organizations, in addition to the concern for workers’ health and well-being, there were huge economic costs because the occurrence of violence leads to the loss of working days for workers and the loss of their ability to work and also increases the willingness of HCWs to leave (Eneroth et al., 2017). Secondly, violence might have a negative impact on the quality of medical care (Arnetz & Arnetz, 2001), which causes HCWs to question social values and norms. Violent behavior could cause work stress and increase the risk of burnout among HCWs (Zhang, 2019). Therefore, understanding the overall violence situation and its associated factors is essential for designing tailored interventions to address this growing problem.

WPV’s multi-level drivers include communication failures, environmental stressors, and institutional gaps. Environmental factors (e.g., overwork, long waits, crowding) directly trigger violence (Shafran-Tikva et al., 2017a; Lau et al., 2012a, 2012b). Patient-related drivers encompass illness-induced fear/anxiety (Mishra, 2015), unrealistic expectations (Xie et al., 2017), treatment dissatisfaction (Carmi-Iluz et al., 2005), poor compliance (Al Ubaidi, 2018), and dependency on HCWs (Kowalczuk & Krajewska-Kułak, 2017; Granek et al., 2019). Critically, communication failures, such as rudeness, inadequate information exchange, miscommunication, or mistrust, significantly elevated violence risk (Hahn et al., 2008; Baig et al., 2018; Edward et al., 2016; Perkins et al., 2017; Mishra, 2015; Joshi & Joshi, 2018; Shi et al., 2015). A key research gap persists, and Duxbury and Whittington (2005) identified a fundamental difference between HCW and patient perceptions regarding the root causes of violence and appropriate staff responses, yet interventions rarely address this discord. Organizationally, low HCW job satisfaction is correlated with higher violence exposure, while improved well-being aids prevention (Berlanda et al., 2019). Recent studies emphasized that the impacts of WPV vary by psychosocial context and individual vulnerability (Dopelt et al., 2022).

Effective prevention requires dual shifts, from reactive HCW training to perpetrator-focused strategies and from generic interventions to targeted approaches for high-risk groups (Spelten et al., 2020a). Chinese research leverages international frameworks to build systemic WPV prevention frameworks (Zhang, 2019), proposing tools like the 6P-approach (public health emergency response, prompt learning from lessons, proactive measures of occupational health, precaution strategies against occupational hazards, personal protective equipment and medical devices supply, and professional networking) for HCW protection during crises (Zhang & Kim, 2021). However, Liu et al. (2017) highlighted a critical implementation gap, and policies must be contextually adapted to China’s unique healthcare dynamics at both the governmental and institutional levels, alongside cultivating a “zero tolerance” occupational culture, a challenge requiring further empirical validation.

This study aims to explore the influencing factors of violence using a comprehensive strategy of quantitative and qualitative study methods to gain a more comprehensive insight. First, the quantitative study focuses on a statistical analysis of the current situation of HCWs suffering from WPV, including its frequency, types, and influencing factors. Second, the qualitative study involves in-depth interviews with HCWs, patients, and their families to uncover different perspectives on WPV from both sides, examining the adverse effects of WPV, its causes, personal coping strategies, and reporting procedures. It also reveals the respondents’ views on methods for preventing and controlling WPV, as well as their suggestions for improving the work environment and enhancing the well-being of HCWs. This study addressed a critical gap by incorporating HCW and patient perspectives, revealing that WPV perceptions vary due to differing expectations and experiences

Therefore, we proposed the following hypotheses:

**H1:**  *Specific occupational roles and work context are associated with a higher risk of WPV.*

**H2:**  *Perceptions about WPV differ between HCWs and patients, leading to contrasting attributions of its causes.*

**H3:**  *A systematic strategy can be developed through this mixed-methods study.*

## 2. Materials and Methods

### 2.1. Quantitative Methods

A cross-sectional survey was conducted in a public hospital in southern China (December 2022~10 January 2023) using cluster sampling. The hospital serves as a regional hub for infectious disease diagnosis/treatment, specializing in tuberculosis, HIV/AIDS, and hepatitis while addressing healthcare challenges within Guangxi’s ethnically diverse and economically constrained population. Since 2015, it has implemented the WHO/ILO HealthWISE tools to enhance occupational health, established an OSH committee, standardized infectious disease protocols, and strengthened staff training. As a designated COVID-19 treatment center in 2020, it integrated HealthWISE principles into its safety culture. This study included 880 HCWs with informed consent.

The quantitative part of this study was conducted using a structured questionnaire developed by the WHO, ILO, ICN, and PSI, including the National Case Study Tool for WPV in Healthcare Settings-Questionnaire (International Labour Office et al., 2003), which investigated the demographic characteristics of all HCWs in the hospital and the occurrence of WPV over the past 12 months. The questionnaire was distributed via electronic communication tools, and the subjects completed the online survey. This questionnaire has been used multiple times for WPV surveys and has good reliability and validity (Acququadro Maran et al., 2018; Magnavita & Heponiemi, 2011; Ferri et al., 2016). Cronbach’s α coefficient was 0.83 (Chen et al., 2019).

Inclusion criteria included the following: (a) healthcare professionals with relevant qualifications; (b) voluntary participation with informed consent; (c) regular employees who have been employed for over one year; and (d) trainees, interns, or students that have been at the hospital for at least six months. Exclusion criteria included the following: (a) absence from duty for over one month during the investigation period and (b) failure to complete the questionnaire in the opening hours. The hospital departments responsible for recruiting HCWs distributed the survey link to qualified participants, and our team performed a rigorous verification of all participants meeting the inclusion and exclusion criteria before data analysis. Participation was voluntary, and an informed consent statement was presented on the first page of the questionnaire.

Categorical variables were analyzed using Chi-square tests with Yates’ continuity correction or Fisher’s exact test for small sample sizes (expected cell count < 5). A binary logistic regression model identified the predictors of violence (with physical violence and psychological violence (verbal abuse, bullying/mobbing, sexual harassment, racial discrimination) as the dependent variable and demographic characteristics, violence attention, etc., as independent variables) and calculated the odds ratio (OR) and 95% confidence interval (CI).

### 2.2. Qualitative Methods

The qualitative part of this study was conducted using semi-structured interviews, where the interview study was carried out according to a self-designed interview outline, targeting HCWs and patients or their families, and the formal interview began after all the respondents gave informed consent. The sample for the qualitative interviews was purposely selected to represent a diverse range of perspectives, which were then transcribed and translated into English. Opinions were collected from HCWs, department leaders, patients, and their families on the trends in the incidence of WPV, causes, and effective measures currently in place at hospitals, and suggestions for future improvements were noted. Interviews were stopped when data saturation (information saturation) was reached. The original recording data was first transcribed into text, and then the raw data was imported into NVivo Pro 12. Two independent researchers conducted iterative coding using NVivo Pro 12. Each part included in the results was obtained with permission from the respondent, anonymized, and edited for grammar and spelling. Then, the content with research value was accurately extracted, and refined coding, classification, and reclassification were carried out to extract the core themes of the interviews. Comprehensive consideration was given to previous studies and international practices, and finally, in-depth discussion and analysis were carried out.

The interview study was conducted through the following self-designed interview outline.
**Theme****HCWs****Patients/Families**Experience and Perception of WPVHave you experienced or witnessed workplace violence in the past year? (If yes, please describe.)Have you experienced or witnessed violence by HCWs against patients? (If yes, please describe.)Consequences of WPVWhat do you think the adverse effects of violent incidents are?What do you think the adverse effects of violent incidents are?Triggers and Attribution1. What do you think the main causes of physical and psychological violence are? 2. In what areas do healthcare institutions need to improve to reduce violence?1. How do you define “violence in healthcare settings”? Please give examples. 2. What do you think the main causes of physical and psychological violence are? 3. How do you view the current tension in doctor–patient relationships in society?Prevention Measures and Effectiveness Evaluation1. What measures has the hospital implemented to prevent violence since July 2021? 2. How do you evaluate the effectiveness of these measures?1. How do you think hospitals are protecting patients and HCWs? 2. What areas of improvement should hospitals prioritize?Response Strategies and Improvement Suggestions1. How do you prevent or respond to violence according to your job responsibilities? 2. Are you willing to report violent incidents? What suggestions do you have for improving the reporting process?1. What action would you take if you were involved in a violent incident? 2. What support do you expect hospitals to provide to help victims?


### 2.3. Connecting Quantitative and Qualitative Findings by Narrative Link

This study adopts a Narrative Link method to deeply integrate quantitative and qualitative data. Its purpose is not only to mutually verify but also to explain and enrich the statistical patterns presented in quantitative analysis through the vivid narratives obtained from qualitative interviews.

## 3. Results

### 3.1. Quantitative Findings

A total of 675 questionnaires were collected; the response rate was 76.7%. All participants are of Chinese nationality. Table 1 compares the relationship between different demographic characteristics (gender, age, ethnicity, marital status, education level, occupation, title, and years of service) and WPV incidence based on survey data from 675 HCWs. The results show that the overall incidence of physical violence is 2.2% (15/675), with higher rates among nurses (3.4%, *p* = 0.034). For psychological violence, verbal abuse has the highest incidence (32.6%, 220/675), with significant risks for females (35.0% vs. males 25.0%, *p* = 0.019) and in outpatient departments (44.1%, *p* < 0.001).

### 3.2. Multifactorial Analysis of WPV

A binary logistic regression model was constructed to analyze the factors influencing WPV, considering whether individuals have experienced physical or psychological violence as dependent variables and the demographic characteristics of HCWs, their level of concern about WPV, and their understanding of methods for responding to violence as independent variables. For physical violence, those with 11~15 years of service have a significantly higher risk (OR = 9.39; 95% CI = 1.14~77.22; *p* = 0.037). For psychological violence, temporary staff (OR = 1.72; 95% CI = 1.21~2.44; *p* = 0.002), direct patient contact (OR = 9.03; 95% CI = 3.21~25.41; *p* < 0.001), and the level of concern about WPV (*p* < 0.001) are independent risk factors. Every increase in the level of concern about WPV is associated with a certain level of higher risk of psychological violence (Table 2).

### 3.3. Timing, Circumstances, and Response to WPV

Table 3 shows the composition of perpetrators, handling methods, and satisfaction levels for different types of violent incidents. Patients are the primary perpetrators, being responsible for 66.7% of physical violence and 55.6% of sexual harassment incidents. Meanwhile, family members are often involved in verbal abuse (40.9%) and bullying (28.6%). Over 80% of violent incidents occur within medical institutions (80.0% physical violence, 85.7% bullying).

### 3.4. Qualitative Findings

#### 3.4.1. Basic Information About the Interviewees

In this study, 28 HCWs (response rate 80.0%) and 17 patients/families (response rate 85.0%) were interviewed using convenient sampling (Table 1).

#### 3.4.2. Main Findings

From the total sample, 28 HCWs and 17 patients/families were also interviewed with open-ended questions. The qualitative analysis concerning WPV against HCWs yielded four themes: theme 1 was “ consequences of WPV”, theme 2 was “defense mechanism”, theme 3 was “causes of WPV”, and theme 4 was “improvement measures” (Table 4).

## 4. Discussion

### 4.1. Current Situation and High-Risk Groups of WPV

In this study, the quantitative results show that the incidence of physical violence is 2.2%, while the incidence of psychological violence is as high as 34.1%, with verbal abuse being the primary form. Further analysis reveals that during the survey period, the overall incidence of physical violence among HCWs was relatively low, but it was higher for specific groups such as men, ethnic minorities, unmarried individuals, nurses, and internal medicine HCWs, which may indicate that these groups face higher risks in their work environment. Night shifts and situations involving direct physical contact with patients (e.g., cleaning or turning) also correlate with a higher incidence of physical violence, possibly because interactions between workers and patients or family members are more frequent in these scenarios, potentially increasing the likelihood of conflict (Silva & Costa, 2023). HCWs with 11–15 years of service face the highest risk of physical violence (OR = 9.4), which underscores the systemic pressures embedded in China’s medical professional title evaluation system. This career stage typically coincides with intense competition for associate chief physician positions (National Health Commission of the People’s Republic of China, 2021). During this period, HCWs often face heavy clinical workloads, stringent performance assessments, and expectations for research output and promotion, all of which may heighten stress levels and increase exposure to patient conflict and violence. Psychological violence incidents are primarily concentrated among female HCWs and emergency departments (*p* < 0.001). Additionally, the results regarding psychological violence show that employment methods, direct patient contact, and concerns about WPV significantly influence experiences of psychological violence. Temporary staff may be more susceptible to psychological violence (Canova-Barrios et al., 2022). This may be because their job status is relatively unstable and low, making them susceptible to unfair treatment or bullying from others, lacking long-term career security, thus making them more likely targets of psychological violence. At the same time, contact with patients can increase tensions and conflicts between them. Moreover, increased concern about WPV is positively correlated with the risk of experiencing psychological violence; the higher the concern about violence, the greater the likelihood of experiencing psychological violence. This could be due to medical professionals’ worries about WPV potentially leading to anxiety and stress, which in turn affects the quality of medical services, treatment outcomes, and individual coping abilities, as well as the harmony of the work environment, thereby triggering violence. These findings highlight the role of the work environment and individual factors in WPV, providing important references for relevant institutions to develop effective prevention strategies. Further research can explore more potential influencing factors and develop targeted intervention measures to ensure the safety of employees’ work environments and their mental health.

Regarding the time and space distribution, 80% of WPV incidents occur within medical institutions, primarily involving patients and their families. Qualitative evidence suggests that direct conflicts and public pressure may trigger psychological violence. For instance, nurses mention that “patients tend to resort to physical force when they don’t understand,” while midwives state that “negative online comments drive me to the brink.” Meanwhile, HCWs emphasize that “emergency department patients have the most unstable emotions,” and nurses describe that “night shifts with family members add significant stress.” This highlights the unique nature of high-risk departments. Patient families complain about “long queues in the morning leading to frequent conflicts,” and administrative staff point out that “small examination rooms exacerbate crowding,” indicating that environmental and temporal factors combined increase the risk of WPV.

### 4.2. Multi-Level Interaction of Violence Causes

WPV is a product of individual behavior, system flaws, and social conflicts, with cognitive gaps between HCWs and patients exacerbating this complexity. At the individual level, differences in expectations lead to behavioral divergence. HCWs view WPV as “part of professional risk,” emphasizing institutional protection and communication training. This aligns with the quantitative findings that highlight that HCWs with direct patient contact have a significantly higher risk of violence (OR = 9.0, *p* < 0.001).

Patients’ emotional distress, high service expectations, and heightened rights awareness are frequent triggers. The qualitative findings show that violence is often linked to “information opacity” and “perceived indifference,” such as a lack of communication or eye contact. Patients may express anger due to disease-related stress or concerns about treatment outcomes (Kaur et al., 2020).

In addition, differences in educational level, cultural background, and values may shape the perceptions of WPV (Khiyani et al., 2023). Patients may resort to complaints or threats to push for better service, while HCWs focus on safety-first protocols. This tension reflects a power imbalance, where HCWs hold clinical authority, and patients respond with informal pressure (Beisecker, 1990).

At the organizational level, delays, overcrowding, and impersonal encounters are common complaints, echoing quantitative data showing higher violence rates in outpatient and emergency departments (44.14%). A patient’s family member noted, “We waited for hours without updates,” reinforcing the link between institutional bottlenecks and violent incidents. Heavy workloads and unclear procedures also contribute to WPV. One nurse remarked, “Who would still want to stay in this job?”, highlighting emotional fatigue. These themes align with survey data linking night shifts and direct contact to elevated violence risk.

The COVID-19 pandemic further intensified WPV by increasing psychological stress for both HCWs and patients. Quantitative data show that HCWs concerned about WPV were over six times more likely to experience it (OR = 6.72). The pandemic heightened psychological stress, impaired communication, and triggered public mistrust. Hossain et al. (2020) noted that such pressures reduce HCWs’ emotional stability and increase patient aggression during crises. The pandemic also worsened social pressures such as isolation and financial hardship. A family member said, “These past few years have been so hard on the family,” capturing the broader context. Resource shortages and media stigmatization further eroded trust, contributing to the “defensive medicine–rights protection violence” cycle (Lorenc et al., 2024).

### 4.3. Optimal Path of Prevention and Control Strategy: CARE Framework Practice

As a global occupational health challenge, the prevention and control of WPV need to break through the traditional single-dimensional path of “security reinforcement” and shift towards systematic interventions centered on strengthening the construction of an occupational health system. The study, based on a mixed-methods approach, proposes the CARE (Communication, Advocacy, Respect, Education) framework, which constructs strategies for violence prevention and occupational health promotion from four dimensions: doctor–patient communication, rights balance, resource allocation, and service optimization.

#### 4.3.1. Communication and Collaboration

The core of occupational health lies in optimizing the work environment and interaction patterns to reduce risks faced by workers. This study found that poor communication is the primary cause of violence, consistent with Duxbury and Whittington theory of the “cognitive gap between healthcare providers and patients (Duxbury & Whittington, 2005). In terms of occupational health, it is necessary to reconstruct the communication ecosystem through the following approaches: introducing the SBAR (Situation–Background–Assessment–Recommendation) standardized communication tool (Shahid & Thomas, 2018); combining scenario simulation training to enhance medical professionals’ non-violent communication skills in high-pressure environments (e.g., empathetic expressions like “I understand your anxiety”); and strengthening two-way information transparency by adopting the UK NHS “informed sharing” model, using visual flowcharts, multilingual manuals, and real-time updates on treatment progress to reduce conflicts caused by information asymmetry. In qualitative interviews, patients mentioned feelings of frustration due to waiting for two hours after an appointment, indicating that “time management commitment” should be included in communication. Organizations should institutionalize conflict resolution by establishing a department for “patient–doctor relationship coordinators”, drawing on a Japanese hospital “third-party mediation committee” mechanism to intervene in the early stages of conflict to prevent escalation.

#### 4.3.2. Advocacy and Autonomy

Occupational health not only focuses on the safety of HCWs but also needs to recognize the rights and interests of patients as “health collaborators”. In this context, the contradiction between patients’ heightened expectations and awareness of their rights and HCWs’ authoritative status and patients’ potential sense of powerlessness may exacerbate conflicts (Beisecker, 1990). The power imbalance between patients and HCWs often leads to different strategies when managing WPV. Implementing joint training programs to foster a mutual understanding of WPV, balance power dynamics, and ensure patients feel heard and respected is crucial. On the other hand, emotional responses and socio-cultural factors also play significant roles in WPV. To alleviate these emotional responses, it is suggested that HCWs be provided with resources for emotional management and stress relief while offering patients emotional support and disease education (Ladak et al., 2023). Additionally, measures for preventing and intervening in WPV should consider cultural diversity and social context, ensuring that strategies are sensitive and adaptable.

Patients’ rights should be publicized in the clinic and waiting area, and the boundaries of medical services and reasonable channels for patients to appeal should be clarified to avoid conflicts caused by “excessive rights protection” (e.g., the practice of Ontario’s Medical Service Quality Act) (Government of Ontario, 2010). In promoting the SDM (Shared Decision Making) Toolkit via decision support cards (e.g., risk/benefit comparison of treatment options), patient engagement increased by 27.0%, and doctor–patient communication conflict decreased by 19.0~26.0% (Durand et al., 2015).

#### 4.3.3. Respect and Resources

Occupational health management needs to advance both “hard resource” allocation and “soft culture” cultivation simultaneously. The under-reporting of “ethnic discrimination” (0.6%) and “sexual harassment” (1.3%) in violent incidents reflects the cultural accumulation of implicit discrimination in medical settings. In 33.3% of physical violence cases, investigations were not initiated, and “verbal warnings” were the primary response method (50.0~74.2%). First, a positive workplace culture should be created to promote respect, empathy, and open communication. Workers should be encouraged to speak up and report any incidents of violence or harassment, and managers should be encouraged to lead by example, responding swiftly when WPV events occur. An “accountability-free” attitude should be adopted towards reports from HCWs, prioritizing the well-being of the workforce (Stacey et al., 2017), and priority should be given to intervening in psychological violence. Secondly, for high-risk departments such as emergency rooms, protective measures should be strengthened, implementing a two-person shift system, night shifts with security personnel, and real-time alerts through intelligent surveillance systems. Finally, according to the consensus of both doctors and patients, it is important to optimize time and space management, and it is suggested to add temporary windows during peak hours (7:00~13:00), changing the clinic layout to “single-channel diversion” to reduce crowd gatherings and thereby lower the risk of violent incidents. The “outpatient-to-emergency diversion algorithm” should be adopted, using AI to predict waiting times and dynamically adjust resource allocation. The implementation of “paperless triage” through the mobile pre-filling of medical history and real-time call number push reduces anxiety caused by cumbersome procedures.

At the institutional level, “anti-discrimination response” should be incorporated into performance evaluations (Yusoff et al., 2023), establishing a closed-loop mechanism of “anonymous reporting–rapid investigation–transparent feedback.” The differences in reporting and feedback mechanisms are also a key issue in the handling of WPV. HCWs may be reluctant to report incidents of violence due to concerns about professional consequences or fear that they cannot resolve the issues after reporting, while patients may choose silence due to a lack of effective feedback channels or distrust in the healthcare system. A long-term tracking and evaluation mechanism should be established, involving regular surveys and interviews with HCWs to build a database of violence cases (Berger et al., 2024). Actively aligning the expectations of both healthcare providers and patients to make them more realistic and achievable and improving the quality and efficiency of medical services to meet patients’ reasonable expectations is an important strategy for reducing WPV. The government, in collaboration with medical institutions, must establish a robust legal and policy framework to address WPV. This includes formulating clear laws, imposing strict sanctions on perpetrators, and providing effective legal protection and support for healthcare personnel (Sergeant & Laws-Chapman, 2012; Hinsenkamp, 2013).

#### 4.3.4. Education and Efficiency

Employers should take primary responsibility for the prevention of WPV and pay attention to the mental health of HCWs (Phillips, 2016), including establishing a safe working environment, providing training, and formulating response strategies. The hospital in question has achieved significant results in enhancing safety management. First, it set up a “Safe Hospital” police office at the entrance and equipped it with an inspection system to ensure the safety and stability of the hospital environment. At the same time, one-key alarm devices were installed in the departments to enable rapid response in emergencies. In addition, the hospital implemented a dual-channel system for medical staff and patients, providing a safer and more orderly passage environment for both parties.

Individuals can improve their psychological endurance by learning psychological adjustment skills, undergoing psychological counseling and guidance sessions, participating in team activities, and other methods (Yeh et al., 2020). In addition, support from colleagues and leaders has a positive effect on reducing workload, work–family conflict, and other negative emotions (Gillet et al., 2018). Secondly, the mental health education and training of medical staff should be strengthened to improve their ability to cope with stress (Qaiser et al., 2015). Workers should be encouraged to practice stress reduction techniques, such as mindfulness, meditation, deep breathing, and physical exercise. Workers should be provided with resources to manage stress, such as counseling services or mental health support; stress management training programs should be implemented; and workers should receive help with developing coping strategies (Ansari, 2024). MBSR (mindfulness-based stress reduction) group courses should be introduced, combined with biofeedback technology to monitor stress levels (Janssen et al., 2018). In addition, employers and organizations should also pay attention to workers’ work pressure and job satisfaction, appropriately adjust work tasks and working hours, and improve work treatment and well-being to reduce the psychological burden of employees and improve their work performance (Hülsheger et al., 2013). Studies have explored the relationship between the job demand–control model and mental health, and multi-dimensional methods such as reducing job demands, enhancing decision-making freedom, and improving the working environment can prevent burnout and emotional exhaustion (Parent-Lamarche et al., 2021; Y.-H. Huang et al., 2011). Flexible work–rest schedules, scheduling, and shift times, as well as night shifts, should be offered (Chiang et al., 2022). To ensure that workers do not overwork themselves, they should be encouraged to maintain a healthy work–life balance. Managers are encouraged to respect HCWs’ private time, avoid contact outside working hours, and provide resources to help workers balance work and life, such as flexible childcare arrangements and parental leave policies (Runze et al., 2023). At the same time, the quality of medical services should be improved.

According to the results of qualitative interviews, the hospital implemented a series of measures during the outbreak to protect the health of medical staff, including priority vaccination, booster shots, the provision of traditional Chinese medicine for prevention, and free chest CT scans (Y. Huang et al., 2024). However, this study still suggests that systematic measures should be taken to help HCWs cope with public health emergencies, enhance risk communication and psychological education, provide accurate epidemic information and preventive measures, and conduct psychological education and training on response strategies (Amde et al., 2024). In addition, the working environment should be improved, a reasonable workload and rest time should be ensured, and the necessary personal protective equipment (PPE) should be provided (National Health Commission of the People’s Republic of China, 2020). Healthy sleep habits should be encouraged, and appropriate rest facilities should be provided for night shift workers. The comprehensive implementation of these measures is expected to reduce the impact of public health emergencies on the mental health of HCWs, improve their well-being, and ensure the quality of medical services and social public health safety (General Office of the CPC Central Committee & General Office of the State Council, 2023).

Through the CARE framework, communication empowerment, cultural restructuring, and process innovation are deeply integrated to build a prevention and control system oriented by the community of a shared future between HCWs and patients. This is not only about the occupational safety of HCWs but also the ethical imperative of achieving universal health coverage (UHC).

### 4.4. Strengths and Limitations

This study conducted an empirical investigation of a certain infectious disease hospital, which has a certain degree of credibility and applicability. This study has the following limitations: (1) the sample is concentrated in a single infectious disease hospital, and the conclusions should be interpreted with caution, which limits transferability not only geographically but also in terms of organizational culture; (2) the interaction effects among different dimensions of the CARE framework were not quantitatively analyzed. Future research could incorporate longitudinal designs to track psychological outcomes or evaluating intervention strategies in diverse clinical settings. Additionally, exploring the co-evolutionary path between WPV prevention and control and healthcare quality improvement based on the “Occupational Health–Patient Safety” synergy model will be an important direction.

## 5. Conclusions

This study highlights the complex, multi-level nature of WPV in healthcare, shaped by individual, organizational, and societal factors. The findings suggest that WPV is not solely driven by individual behaviors but is also influenced by structural and relational dynamics, with organizational stressors and societal pressures, particularly the COVID-19 pandemic, exacerbating violence. For practitioners, this study emphasizes the need for joint training programs and systemic organizational reforms that address both HCWs’ and patients’ needs. Clear communication, emotional regulation, and respect-building measures are key to reducing violence and improving care environments. The CARE framework offers a promising approach to mitigating WPV. Future research should focus on longitudinal studies to track psychological outcomes or evaluate intervention strategies in diverse clinical settings to assess the effectiveness of the CARE model over time and its adaptability across different healthcare contexts.

## Figures and Tables

**Table 1:** The socio-demographic characteristics of the sample (N = 675).

	Total (N = 675)	Physical Violence (n = 15)		Psychological Violence (n = 289)							
				Verbal Abuse (n = 220)		Bullying/Mobbing (n = 56)		Sexual Harassment (n = 9)		Ethnic Discrimination (n = 4)	
		n (%)	*p*	n (%)	*p*	n (%)	*p*	n (%)	*p*	n (%)	*p*
Gender			0.375		0.019		0.161		0.916		0.951
man	160 (23.7)	5 (3.13)		40 (25.0)		9 (5.6)		2 (1.3)		3 (1.9)	
woman	515 (76.3)	10 (1.94)		180 (35.0)		47 (9.1)		7 (1.4)		1 (0.2)	
Age (years)			0.778		0.094		0.033		0.112		0.255
≤25	24 (3.6)	0 (0)		6 (25.0)		1 (4.2)		0 (0)		0 (0)	
26~	280 (41.5)	6 (2.1)		84 (30.0)		13 (4.6)		0 (0)		0 (0)	
36~	252 (37.3)	7 (2.8)		90 (35.7)		29 (11.5)		6 (2.4)		2 (0.8)	
46~	82(12.1)	2 (2.4)		33 (40.2)		10 (12.2)		2 (2.4)		1 (1.2)	
56~	37 (5.5)	0 (0)		7 (18.9)		3 (8.1)		1 (2.7)		1 (2.7)	
Nationality			0.569		0.252		0.127		0.13		0.126
Han	319 (47.3)	6 (1.9)		97 (30.4)		21 (6.6)		2 (0.6)		0 (0)	
ethnic minorities	356 (52.7)	9 (2.55)		123 (34.6)		35 (9.8)		7 (2.0)		4 (1.1)	
Marriage			0.712		0.484		0.201		0.392		0.388
unmarried	192 (28.4)	5 (2.6)		58 (30.2)		11 (5.7)		1 (0.5)		0 (0)	
married	459 (68.0)	10 (2.2)		152 (33.1)		44 (9.6)		8 (1.7)		4 (0.9)	
other	24 (3.6)	0 (0)		10 (41.7)		1 (4.2)		0 (0)		0 (0)	
Degree of education			0.534		0.834		0.641		0.755		1
college and below	126 (18.7)	4 (3.2)		40 (31.8)		13 (10.3)		2 (1.6)		1 (0.8)	
undergraduate college	479 (70.9)	9 (1.9)		155 (32.4)		37 (7.7)		7 (1.5)		3 (0.6)	
Master’s degree or above	70 (10.4)	2 (2.9)		25 (35.7)		6 (8.6)		0 (0)		0 (0)	
Occupation			0.034		<0.001		0.043		0.466		0.044
medical technology and administration	165 (24.4)	0 (0)		34 (20.6)		8 (4.9)		2 (1.2)		1 (0.6)	
doctor	184 (27.3)	4 (2.2)		68 (37.0)		17 (9.2)		4 (2.2)		3 (1.6)	
nurse	326 (48.3)	11 (3.4)		118 (36.2)		31 (9.5)		3 (0.9)		0 (0)	
Professional titles			0.117		<0.001		0.002		0.006		0.019
senior	265 (39.3)	4 (1.5)		56 (21.1)		19 (7.2)		5 (1.9)		3 (1.1)	
middle rank	240 (35.6)	2 (0.8)		76 (31.7)		20 (8.3)		4 (1.7)		1 (0.4)	
elementary	115 (17.0)	9 (7.8)		76 (66.1)		12 (10.4)		0 (0)		0 (0)	
none	55 (8.1)	0 (0)		12 (21.8)		5 (9.1)		0 (0)		0 (0)	
Length of service (years)			0.040		0.799		0.840		0.114		0.497
0~5	178 (26.4)	1 (0.6)		59 (33.2)		15 (8.4)		1 (0.6)		0 (0)	
6~10	178 (26.4)	2 (1.2)		62 (34.8)		16 (9.0)		2 (1.1)		1 (0.6)	
11~15	139 (20.6)	7 (5.0)		47 (33.8)		13 (9.4)		5 (3.6)		2 (1.4)	
16~20	79 (11.7)	1 (1.3)		23 (29.1)		4 (5.1)		0 (0)		0 (0)	
≥21	101 (14.9)	4 (4.0)		29 (28.7)		8 (7.9)		1 (1.0)		1 (1.0)	
Appointment			0.540		<0.001		<0.001		0.008		0.237
full-time	302 (44.7)	7 (2.3)		119 (39.4)		38 (12.58)		8 (2.65)		3 (0.99)	
temporary staff	373 (55.3)	8 (2.1)		101 (27.1)		18 (4.83)		1 (0.27)		1 (0.27)	
Monthly income (CNY/month)			0.565		0.028		0.393		0.917		0.394
<4000	206 (30.5)	4 (1.94)		53 (25.7)		13 (6.3)		2 (1.0)		0 (0)	
4000~	215 (31.9)	5 (2.33)		72 (33.5)		18 (8.4)		3 (1.4)		1 (0.5)	
6000~	254 (37.6)	6 (2.36)		95 (37.4)		25 (9.8)		4 (1.6)		3 (1.2)	
Department			0.325		0.001		0.257		0.816		0.638
administration	95 (14.1)	1 (1.1)		18 (19.0)		8 (8.4)		1 (1.1)		0 (0)	
medicine	266 (39.4)	9 (3.4)		99 (37.2)		25 (9.4)		2 (0.8)		1 (0.4)	
surgery	52 (7.7)	2 (3.9)		17 (32.7)		3 (5.8)		1 (1.9)		1 (1.9)	
medical technology	151 (22.4)	1 (0.7)		37 (24.5)		7 (4.6)		3 (2.0)		1 (0.7)	
outpatient and emergency	111 (16.4)	2 (1.8)		49 (44.1)		13 (11.7)		2 (1.8)		1 (0.9)	
Shift work			0.218		0.002		0.094		0.167		0.453
yes	554 (82.1)	14 (2.5)		195 (35.2)		50 (9.0)		9 (1.6)		4 (0.7)	
no	121 (17.9)	1 (0.8)		25 (20.7)		6 (5.0)		0 (0)		0 (0)	
Night job			0.058		0.082		0.568		0.059		0.286
yes	494 (73.2)	14 (2.8)		169 (34.2)		41 (8.3)		9 (1.8)		4 (0.8)	
no	181 (26.8)	1 (0.6)		51 (28.2)		15 (8.3)		0 (0)		0 (0)	
Interact directly with patients			0.001		<0.001		0.018		0.293		0.464
Yes (directly)	391 (57.9)	15 (3.8)		159 (40.7)		35 (9.0)		8 (2.1)		4 (1.0)	
Yes (not direct)	189 (28.0)	0 (0)		57 (30.2)		20 (10.6)		1 (0.5)		0 (0)	
No	95 (14.1)	0 (0)		4 (0)		1 (0)		0 (0)		0 (0)	
Number of workers working together			0.845		0.247		0.165		0.115		0.032
N/A	7 (1.0)	0 (0)		4 (57.1)		2 (28.6)		1 (14.3)		1 (14.3)	
<5	111 (16.5)	3 (2.7)		32 (28.8)		6 (5.4)		2 (1.8)		1 (0.9)	
5~	170 (25.2)	4 (2.4)		50 (29.4)		15 (8.8)		2 (1.2)		0 (0)	
16~	387 (57.3)	8 (2.1)		134 (34.6)		33 (8.5)		4 (1.0)		2 (0.5)	
Concerns about WPV			0.206		<0.001		<0.001		0.215		0.023
no worries at all	200 (29.6)	5 (2.5)		22 (11.0)		5 (2.5)		1 (0.5)		0 (0)	
no worries	167 (24.8)	3 (1.8)		46 (27.5)		10 (6.0)		1 (0.6)		0 (0)	
uncertain	191 (28.3)	2 (1.1)		85 (44.5)		19 (10.0)		4 (2.1)		2 (1.1)	
worried	52 (7.7)	1 (1.9)		30 (57.7)		11 (21.2)		2 (3.9)		2 (3.9)	
very worried	65 (9.6)	4 (6.2)		37 (56.9)		11 (16.9)		1 (1.5)		0 (0)	
WPV prevention training			0.908		0.207		0.049		0.779		0.411
yes	578 (85.6)	13 (2.3)		183 (31.7)		43 (7.4)		8 (1.4)		4 (0.7)	
no	97 (14.4)	2 (2.1)		37 (38.1)		13 (13.4)		1 (1.0)		0 (0)	

**Table 2:** Multivariate logistic regression analysis of OR values of physical and psychological violence and potential risk factors among HCWs.

	β	SE	Wald χ^2^	*p*	OR (95%CI)
Physical violence (n = 15)					
Length of service (years)					
0~5					Ref
6~10	0.7	1.2	0.3	0.573	2.0 (0.2~22.4)
11~15	2.2	1.1	4.3	0.037	9.4 (1.1~77.2)
16~20	0.8	1.4	0.3	0.564	2.3 (0.1~36.8)
≥21	2.0	1.1	3.1	0.077	7.3 (0.8~66.2)
Psychological violence (n = 289)					
Appointment method					
full-time					Ref
temporary staff	0.5	0.2	9.1	0.002	1.7 (1.2~2.4)
Direct contact with patients					
no					Ref
yes	2.2	0.5	17.4	<0.001	9.0 (3.2~25.4)
Level of concern about WPV					
no worries at all					Ref
no worries	0.9	0.3	10.0	0.002	2.4 (1.4~4.2)
uncertain	1.5	0.3	31.9	<0.001	4.5 (2.7~7.6)
worry	1.9	0.4	27.7	<0.001	6.7 (3.3~13.6)
very worried	1.9	0.3	30.9	<0.001	6.5 (3.4~12.7)

**Table 3:** Distribution of perpetrators and responses to violent incidents.

Project	Physical Violence (n = 15)		Psychological Violence							
			Verbal Abuse (n = 220)		Bullying/Mobbing (n = 56)		Sexual Harassment (n = 9)		Ethnic Discrimination (n = 4)	
	n	%	n	%	n	%	n	%	n	%
Perpetrator										
patient	10	66.7	102	46.4	31	55.4	5	55.6	3	75.0
family members of the patient	2	13.3	90	40.9	16	28.6	2	22.2	0	0
managers and colleagues	0	0	9	4.1	3	5.3	0	0.00	0	0
the public and others	3	20.0	19	8.6	6	10.7	2	22.2	1	25.0
Setting										
medical institution	12	80.0	186	84.5	48	85.7	6	66.7	3	75.0
at the patient’s home	0	0	18	8.2	7	12.5	0	0.0	0	0
outdoors	1	6.7	11	5.00	0	0.0	1	11.1	0	0
other	2	13.3	5	2.3	1	1.8	2	22.2	1	25.0
Understanding of the cause of the incident										
yes	5	33.3	67	30.5	31	55.4	4	44.4	3	75.0
no	10	66.7	153	69.5	25	44.6	5	55.6	1	25.0
Investigation party										
management/employer	5	100.0	64	95.5	29	93.6	4	100.0	2	66.7
trade union	1	20.0	17	25.4	9	29.0	0	0	2	66.7
association	1	20.0	9	13.4	6	19.4	2	50.0	2	66.7
consciousness of community	1	20.0	11	16.4	6	19.4	0	0	1	33.3
police	1	20.0	18	26.9	12	38.7	3	75.0	3	100.0
other	1	20.0	4	6.0	2	6.5	0	0	0	0
Way of dealing with the attacker										
no process	3	60.0	21	31.3	11	35.5	2	50.0	1	33.3
verbal warning	3	60.0	44	65.7	23	74.2	2	50.0	2	66.7
stop treatment	1	20.0	10	14.9	7	22.6	3	75.0	1	33.3
report to the police	2	40.0	19	28.4	12	38.7	1	25.0	1	33.3
public prosecution	1	20.0	8	12.0	3	9.7	1	25.0	0	0
other	3	60.0	10	14.9	3	9.68	0	0	0	0
Degree of satisfaction with the handling of the incident										
very dissatisfied	3	20.0	13	5.9	9	16.4	2	22.2	0	0
dissatisfied	0	0	39	17.7	12	21.8	1	11.1	1	25.0
neutral	5	33.3	78	35.5	18	32.7	4	44.5	3	75.0
satisfied	4	26.7	53	24.1	10	18.2	0	0	0	0
very satisfied	3	20.0	37	16.8	6	10.9	2	22.2	0	0
Measures provided by the employer										
advice	13	86.7	145	65.9	34	64.3	6	66.7	3	75.0
outlets to talk or report	13	86.7	146	66.4	33	58.9	5	55.6	2	50.0
other support	13	86.7	147	66.8	35	62.5	6	66.7	2	50.0

Note: Perpetrator, incident location, and investigation of causes were single-choice questions; investigation parties, handling of attackers, and employer measures were multiple-choice questions.

**Table 4:** Scope and codes of WPV.

Themes	Categories/Subthemes	Codes	HCWs	Patients/ Families	Total	Citation
1. Consequences of WPV						
	1.1 impact on HCWs	1.1.1 psychological trauma and negative emotions	7	3	10	“Who would still want to do this job if it keeps going like this? Sooner or later, I’ll have to think about changing careers.” —Nurse, Endoscopy Unit “If doctors get scolded too often, their attitude is bound to change. They won’t take our illness seriously anymore.” —Inpatient, Infectious Disease Ward
		1.1.2 threat to personal safety	2	3	5	
		1.1.3 work interference and career obstruction	4	1	5	
		1.1.4 family life impact	3	0	3	
	1.2 impact on patients	1.2.1 compromised medical experience	5	7	12	“You have to protect yourself first before even thinking about how to treat the patient. Sometimes it’s impossible to stay calm and focused.” —Doctor, Pediatrics Department “Violence makes doctors afraid of us. Then they stop caring. Isn’t that hurting us too?” —Patient, Hepatology Outpatient Clinic
		1.2.2 hindered disease recovery	2	7	9	
		1.2.3 mental health deterioration	0	7	7	
		1.2.4 reputational damage to patients/families	0	3	3	
	1.3 social impact	1.3.1 decreased trust between HCWs and patients	4	1	5	“The media always tells just one side of the story—the part where the doctor is at fault.” —Nurse, Obstetrics and Gynecology “So many doctor–patient conflicts are made worse by what gets spread online. Now nobody trusts anyone.” —Family, Infectious Disease Inpatient Ward
		1.3.2 negative societal effects	2	1	3	
2. WPV defense mechanisms						
	2.1 perspectives of both parties	2.1.1 calming patient emotions	9	8	17	“Nowadays, communication skills are crucial. We have to be extra cautious with how we speak.” —Doctor, General Surgery Outpatient Clinic “The first thing is to calm the patient down and stop them from getting agitated.” —Patient, Respiratory Department
		2.1.2 activating alarm systems	8	6	14	
	2.2 patient perspectives	2.2.1 complaints, threats, tolerance, bystanding	0	7	7	“If the doctor’s attitude is bad, I’ll splash my blood on them.” —Patient, HIV Outpatient Clinic
	2.3 HCWs perspectives	2.3.1 self-protection strategies	3	0	3	“We rely on the alarm system and surveillance cameras now, but when something really happens, it’s still just us on our own.” —Nurse, General Surgery Outpatient Clinic
3. Causes of WPV						
	3.1 individual level	3.1.1 perpetrator-related factors				“We had a psychiatric patient suddenly start hitting people and smashing things. It was terrifying.” —Doctor, Infectious Disease Clinic “When you don’t understand the illness or the charges, it’s easy for emotions to explode.” —Family, HIV Clinic
		3.1.1.1 patient emotional instability (mental illness/drug abuse)	9	7	16	
		3.1.1.2 patient misunderstanding of medical services	4	5	9	
		3.1.1.3 unrealistic patient expectations	6	3	9	
		3.1.1.4 heightened patient rights awareness	0	1	1	
		3.1.2 victim-related factors				
		3.1.2.1 poor HCW–patient communication	6	8	14	
		3.1.2.2 inadequate medical service quality	2	3	5	
	3.2 organizational level	3.2.1 workplace issues				“The line was way too long. Some patients just lose it and start yelling.” —Nurse, Radiology Department “Every day there’s a long queue on the first floor, and no one to answer your questions. That kind of thing makes people furious.” —Family, HIV Inpatient Ward
		3.2.1.1 prolonged waiting time	7	2	9	
		3.2.1.2 high-risk department characteristics (HIV/emergency care)	2	0	2	
		3.2.2 physical environment				
		3.2.2.1 inefficient triage/guidance systems	4	1	5	
		3.2.2.2 outdated facilities	0	3	3	
		3.2.3 work design problems				
		3.2.3.1 heavy workload and staff shortages	5	1	6	
		3.2.3.2 lack of institutional violence prevention mechanisms	1	0	1	
	3.3 societal level	3.3.1 public misunderstanding due to media misrepresentation	4	2	6	“When the COVID test results aren’t out, patients accuse us of delaying things. It’s unbearable.” —Nurse, Fever Clinic “These past few years of the pandemic have been so hard on the family.” —Family, Pediatrics Outpatient Clinic
		3.3.2 policy contradictions in healthcare accessibility	3	3	6	
		3.3.3 impact of COVID-19 pandemic	3	1	4	
4. Prevention measures						
	4.1 individual level	4.1.1 enhanced communication and health education	7	3	10	“When I start with ‘I understand how you feel,’ the chances of conflict go down by half.” —Director, Doctor–Patient Communication Office “If doctors just listened to us a bit more, and didn’t act so high and mighty, who would want to get violent?” —Patient, Pediatrics Outpatient Clinic
		4.1.2 improved medical service quality	5	4	9	
		4.1.3 increased empathy and responsiveness	4	2	6	
	4.2 organizational level	4.2.1 enhanced triage/guidance systems	4	1	5	“Now that we have a security guard on night shifts, I feel much safer.” —Nurse, Fever Clinic “With all these people trying to see a doctor, can’t the hospital assign more staff to guide us? Who are we supposed to ask?” —Family, HIV Outpatient Clinic
		4.2.2 increased staffing and rational time allocation	3	2	5	
		4.2.3 improved appointment/queue systems	2	2	4	
		4.2.4 strengthened institutional support (e.g., security)	11	1	12	
		4.2.5 facility upgrades	2	1	3	
		4.2.6 specialized department management (e.g., HIV units)	2	1	3	
	4.3 societal level	4.3.1 national policy adjustments				“We need legal support. There should be explicit criminal laws against violence in medical settings.” —Hospital Administrator “If health insurance could cover more and reduce our burden, that would really help.” —Patient, Hepatology Outpatient Clinic
		4.3.2 improved health insurance policies	2	3	5	
		4.3.3 enhanced violence prevention policies	3	2	5	

## Data Availability

The datasets used and analyzed in this study are available from the corresponding author upon reasonable request.

## Abbreviations

The following abbreviations are used in this manuscript:

WHO	World Health Organization
ILO	International Labor Organization
ICN	International Council of Nurses
PSI	Public Service International
WPV	Workplace violence
HCW	Healthcare worker
PPE	Personal protective equipment
UHC	Universal health coverage
